# Lateral habenula perturbation reduces default-mode network connectivity in a rat model of depression

**DOI:** 10.1038/s41398-018-0121-y

**Published:** 2018-03-27

**Authors:** Christian Clemm von Hohenberg, Wolfgang Weber-Fahr, Philipp Lebhardt, Namasivayam Ravi, Urs Braun, Natalia Gass, Robert Becker, Markus Sack, Alejandro Cosa Linan, Martin Fungisai Gerchen, Jonathan Rochus Reinwald, Lars-Lennart Oettl, Andreas Meyer-Lindenberg, Barbara Vollmayr, Wolfgang Kelsch, Alexander Sartorius

**Affiliations:** 1 RG Translational Imaging, Department of Neuroimaging, Central Institute of Mental Health, Medical Faculty Mannheim, University of Heidelberg, Mannheim, Germany; 2 Department of Psychiatry and Psychotherapy, Central Institute of Mental Health, Medical Faculty Mannheim, University of Heidelberg, Mannheim, Germany; 3 RG Developmental Biology of Psychiatric Disorders, Department of Psychiatry, Central Institute of Mental Health, Medical Faculty Mannheim, University of Heidelberg, Mannheim, Germany; 4 RG Systems Neuroscience in Psychiatry, Department of Psychiatry, Central Institute of Mental Health, Medical Faculty Mannheim, University of Heidelberg, Mannheim, Germany; 5 Institute of Psychopharmacology, Central Institute of Mental Health, Medical Faculty Mannheim, University of Heidelberg, Mannheim, Germany; 6 Department of Clinical Psychology, Central Institute of Mental Health, Medical Faculty Mannheim, University of Heidelberg, Mannheim, Germany; 7 RG Animal Models in Psychiatry, Department of Psychiatry, Central Institute of Mental Health, Medical Faculty Mannheim, University of Heidelberg, Mannheim, Germany

## Abstract

Hyperconnectivity of the default-mode network (DMN) is one of the most widely replicated neuroimaging findings in major depressive disorder (MDD). Further, there is growing evidence for a central role of the lateral habenula (LHb) in the pathophysiology of MDD. There is preliminary neuroimaging evidence linking LHb and the DMN, but no causal relationship has been shown to date. We combined optogenetics and functional magnetic resonance imaging (fMRI), to establish a causal relationship, using an animal model of treatment-resistant depression, namely *Negative Cognitive State* rats. First, an inhibitory light-sensitive ion channel was introduced into the LHb by viral transduction. Subsequently, laser stimulation was performed during fMRI acquisition on a 9.4 Tesla animal scanner. Neural activity and connectivity were assessed, before, during and after laser stimulation. We observed a connectivity decrease in the DMN following laser-induced LHb perturbation. Our data indicate a causal link between LHb downregulation and reduction in DMN connectivity. These findings may advance our mechanistic understanding of LHb inhibition, which had previously been identified as a promising therapeutic principle, especially for treatment-resistant depression.

## Introduction

Converging evidence from human neuroimaging^1,2^ and post-mortem^3^ studies supports the notion that the lateral habenula (LHb) plays a crucial role in major depressive disorder (MDD). Data from patients^2,4^, as well as from rodent models of depression^5,6^ indicate that the LHb is generally overactive in depression, although other studies in both humans^7^ and animals^8^ have suggested a more complex relationship.

Physiologically, the LHb encodes negative reward prediction error, that is, the omission of an expected reward, or the occurrence of an unexpected punishment^9^. Through its efferents, the LHb modulates monoaminergic nuclei, particularly the dorsal raphe^10^ and ventral tegmental area^9,11^. This is a potentially important mechanistic link to depression pathophysiology in light of the monoamine hypothesis of mood disorders.

Furthermore, several lines of evidence indicate that LHb inhibition can be a therapeutic approach for depression: we reported on two patients with treatment-refractory depression in whom deep brain stimulation (DBS) of the LHb was applied successfully^12^. Corresponding to this, LHb DBS rescued electrophysiological abnormalities, as well as depressive-like behavior in a rat model of treatment-resistant depression, congenital learned helplessness (cLH)^6^. Similar behavioral effects were observed in this model following pharmacological inhibition^13^.

On the neural network level, default-mode network (DMN) hyperconnectivity is one of the most consistent findings in human depression^14–16^. It is the anterior DMN that is most reliably altered^14,15,17–21^ (for review, see Mulders et al.^16^), whereas hyperconnectivity of the posterior DMN has also been described^16^, but not unequivocally^16,21–24^. The DMN appears particularly promising for translation between humans and animal models as it has consistently been found across species—in rats, particularly, the DMN is remarkably homologous to the human DMN^25–27^, and the anterior DMN was delineated as a major subnetwork in a hypothesis-free analysis^27^. In light of this and the human depression literature cited above, our primary hypothesis concerned connectivity changes in the anterior DMN. As the medial parts of the posterior DMN (posterior cingulate cortex and precuneus in humans; retrosplenial cortex in rats) have also been widely implicated in human depression studies^23,24,28–31^, and since we previously reported on increased connectivity between anterior and posterior DMN regions (cingulate and retrosplenial cortices) in the same rat model of depression^8^, we also included the retrosplenial cortex in this hypothesis.

There is some limited evidence from human neuroimaging pointing to a connection between the DMN and the habenula: during rest, habenula blood oxygen level dependent (BOLD) activity correlated with several parts of the DMN^1,32^. Importantly, this correlation was associated with subclinical depression scores^1^.

However, no studies to date have investigated a potential mechanistic link between LHb and the DMN. Accordingly, the aim of the current study was to test a possible causal relationship between LHb inhibition and DMN connectivity. LHb inhibition, as detailed above, is a promising therapeutic principle for depressive disorders. DMN hyperconnectivity, on the other hand, is a highly consistent system-level phenotype of depression. To test a link between the two, we used the combination of optogenetics and functional magnetic resonance imaging (fMRI). Optogenetics refers to the transduction of neurons with a light-sensitive membrane protein^33^, leading to depolarization or hyperpolarization upon local laser illumination, and thus stimulation or inhibition, respectively. For the present study, we used archaerhodopsin, an inhibitory opsin. Translational resting-state fMRI has been developed to narrow the bridge between clinical and preclinical findings, thus enhancing drug development^34^.

Our primary hypothesis was that perturbation of LHb activity would lead to decreased DMN connectivity.

## Materials and methods

### Animals

We used the Negative Cognitive State (NC) rat model of depression^35^. The term NC has come to substitute the previous terminology of “cLH”. cLH rats had been bred for high learned helplessness^36^, that is, inescapable shock leading to failure to escape from escapable shock. In later generations, helpless behavior was found even without prior inescapable stress, thereby reducing animal harm^36^. Therefore, we have recently begun to use the term NC, which also reflects the depressive-like cognitive abnormalities present in this model^37^. cLH/NC is a valid model for MDD^37,38^, in particular treatment-resistant depression^39^.

In total, 19 male rats were used. Since this was an exploratory study, no formal power or sample-size estimation was performed. The group size was based on prior experience and literature reports on prior optogenetic-fMRI studies (e.g.,^40–42^).

All animal procedures were approved by the local Animal Welfare Committee (Regierungspräsidium Karlsruhe, Germany) and were in accordance with the regulations on animal research within the European Union (European Communities Council Directive 86/609/EEC) and the German Animal Welfare Act.

### Optogenetics

For optogenetics, we used a rAAV-CaMKIIa-ArchT:GFP viral construct. rAAV denotes the recombinant adenoviral construct. The CaMKIIa promoter leads to specific expression in neurons, with a relative preference for glutamatergic neurons^42^. GFP is a fluorescent protein used for histological control of successful infection. ArchT(3.0) is an inhibitory opsin (archeorhodopsin), whose activation by yellow light leads to proton transport out of the cell, and thus hyperpolarization^43^. To describe this manipulation, we use the conservative term “perturbation” rather than “inhibition”, as the primary inhibition can be followed by rebound effects^44,45^, and prolonged illumination can lead to increased (rather than decreased) spontaneous synaptic activity, although the latter effect has only been observed for axon terminals^44^. In the present study, ArchT-expressing LHb neurons were illuminated predominantly at the cell body.

At 8 weeks of age, rats underwent isoflurane anesthesia and the virus was stereotactically injected bilaterally into the LHb (for details, see Supplementary Methods). Of the 13 rats injected with ArchT, 8 animals fulfilled inclusion criteria, namely bilateral robust GFP expression within, but no significant expression outside of, the LHb. Additionally, six animals (control group) were injected with a virus lacking ArchT. Thus, 14 animals (8 ArchT and 6 controls) were included in further analyses.

Three weeks after virus injection, animals were anesthetized again for bilateral stereotactic implantation of fiber-optic cannulae, which were fixed with dental cement (for details, see Supplementary Methods). Animals recovered at least three days before scanning (mean 4.0; range 3–5 days; matched between groups: *p* > 0.5).

### Laser stimulation

**Fig. 1:**
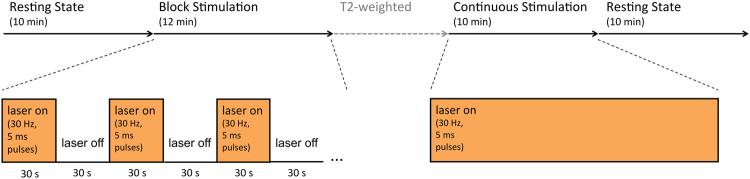
**Sequence of BOLD-fMRI acquisitions and concurrent laser stimulation**

Yellow light (593 nm, see Supplementary Methods) was applied during fMRI acquisition in the following sequence: four BOLD time series were acquired (see Fig. 1). First, a resting-state session of 10 min without any laser stimulation; second, a “block” sequence of 12 min with alternating “laser-on” and “laser-off” periods lasting 30 s each; third, a session of 10 min with continuous laser stimulation; fourth, another resting-state session of 10 min without any laser stimulation. (The high-resolution T2-weighted image was acquired between the second and third session, in order to reduce continuous energy deposition potentially resulting in heating artifacts.) Laser was applied with a frequency of 30 Hz of light pulses with a duration of 5 ms. (This applied both for the “laser-on” periods in the block session and during the session of continuous laser stimulation.) We decided to use a pulsed laser stimulation rather than continuous illumination, in order to minimize energy deposition and thus heating artifacts^44^.

### Scanning procedures

MRI data were acquired with a Bruker Biospec MRI scanner (9.4T; Bruker, Ettlingen, Germany). Anesthesia was started with 4% isoflurane, which was then reduced to 2.5% for scanning preparations. Subsequently, a bolus of 0.08 mg/kg medetomidine was applied. Isoflurane was then tapered off within 10 min, after which a continuous dose of 0.3 mg/kg/h medetomidine was started. fMRI experiments commenced 20 min after isoflurane discontinuation.

Functional data were acquired using a T2*-weighted Echo Planar Imaging sequence with the following parameters: repetition time/echo time = 1.7 s/17.5 ms, 96 × 96 matrix, field of view = 35 × 35 mm², 29 slices (0.5 mm thickness, 0.2 mm gap). See Supplementary Materials for further details on scanning parameters.

### Image data processing

All data were processed using FMRIB Software Library (FSL, version 4.1) (http://www.fmrib.ox.ac.uk/fsl), SPM8 (http://www.fil.ion.ucl.ac.uk/spm/software/spm8/), as well as our own Matlab (http://www.mathworks.com/products/matlab/) scripts. The brain was extracted from three-dimensional (3D) anatomical T2-weighted images. The functional images were corrected for geometrical distortions using the acquired B0-maps and realigned to the brain-extracted T2-weighted anatomical images. Subsequently, a correction for physiological noise was conducted using the Aztec^46^ software employing the RETROICOR^47^ method with the physiological data sampled during the measurements. Functional data were slice-time corrected and normalized to a Paxinos-space digital atlas^48^ using the normalization parameters of the 3D-structural image. For statistical parametric mapping (SPM) analyses, the normalized data were additionally smoothed with a 0.6 mm isotropic Gaussian kernel. For connectivity analyses, the cerebrospinal fluid signal was regressed out, and a bandpass filter (0.01–0.1 Hz) was applied.

### Statistical analysis

#### BOLD activity analysis

As a hypothesis-free analysis, we tested whether LHb perturbation would lead to acute changes in BOLD level. To this end, the block-design imaging sequence was analyzed with a standard general linear model approach. Briefly, the “laser-on” condition was used as the independent variable for a first-level analysis in each animal. For group statistics (ArchT vs. control virus), the resulting beta images were then analyzed in a second-level analysis.

#### Network connectivity analyses

**Fig. 2:**
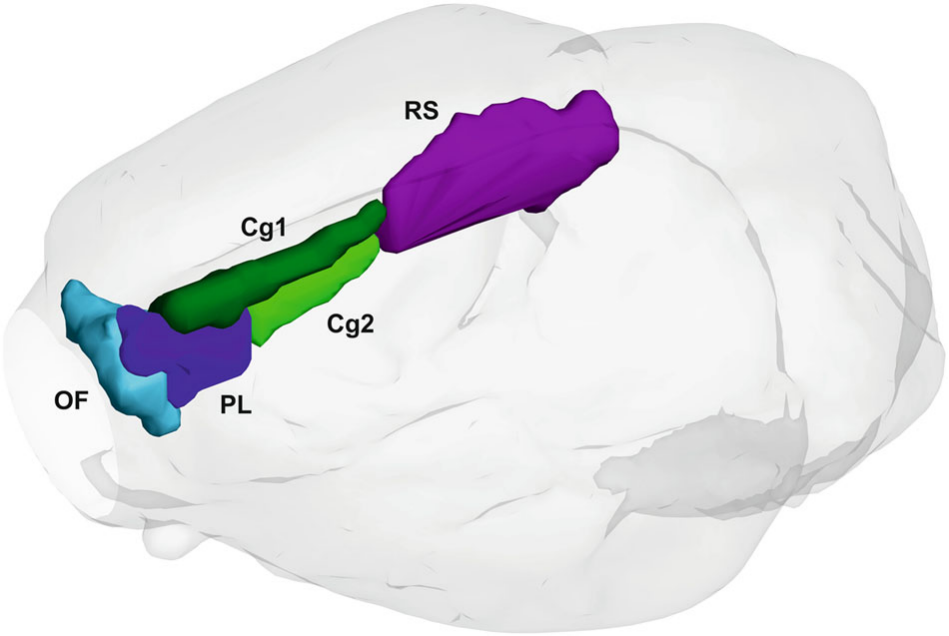
Anatomical representation of the default-mode network (DMN) regions included in the analysis: light blue, orbitofrontal cortex (OF); dark blue, prelimbic cortex (PL); dark green, cingulate cortex 1 (Cg1), light green, cingulate cortex 2 (Cg2); violet, retrosplenial cortex (RS). For clarity, only regions in right hemisphere are shown. Regions are displayed against a semi-transparent brain surface created from the structural MRI template used. Surface of regions was smoothed for better visibility

We chose a network approach for analysis of DMN connectivity changes following LHb perturbation. To define the nodes of the network, we used the rat brain MRI atlas to which the imaging data had been normalized^48^. According to previous work on the DMN in rats^25,26^ and its subnetworks^27^, we selected the following atlas regions, separately for each hemisphere: prelimbic cortex, orbitofrontal cortex, cingulate cortex 1 and 2 (dorsal and ventral part, respectively) and retrosplenial cortex. This yielded a network of 10 nodes; see Fig. 2 for anatomical illustration. Mean BOLD time courses were extracted from these regions, separately for each hemisphere. Then, Pearson’s correlation coefficient was computed pairwise between regional time courses, for each animal. For further analyses, we used Fisher-Z-transformed correlation matrices.

For assessment of acute connectivity changes during the block design, we used network-wide psychophysiological interaction (PPI) analysis^49^. PPI tests whether the temporal correlation between two regional BOLD time courses (“physio”) changes depending on a paradigm (“psycho”, in this case, the laser block sequence). Group comparison of the PPI matrices was then done using the Network-based statistic (NBS) approach^50^, see below for details.

Second, we subtracted the correlation matrices of the third BOLD sequence (continuous laser stimulation) minus the matrices from the first BOLD sequence (resting-state; see Fig. 1). The subtraction matrices were then compared between ArchT and control groups, again using NBS.

Third, to test for connectivity changes that are delayed or maintained, we did an analogous subtraction, but this time between the fourth and the first BOLD sequence.

Briefly, NBS is a non-parametric method for network-wide hypothesis testing. It employs a cluster-based permutation test to control for multiple comparisons. First, for each single edge, a two-sample *t*-test is performed comparing the ArchT and control groups. At this point, no statistical inference is done; only the *t*-value is stored. Next, edges with *t*-values below a certain threshold are discarded. (This threshold was set to *t* = 1.8, corresponding to *p* < 0.05 with 13 degrees of freedom; note that the selection of this threshold still leads to a conservative treatment of the multiple comparison problem since a permutation test is subsequently performed.) Contiguous threshold-surviving edges are defined as a cluster. The intensity of the resulting cluster is noted, that is, the number of contiguous threshold-surviving edges weighted by the *t-*values. Finally, a permutation test compares this intensity with the maximum intensities of 5000 random permutations. As a one-sided test is computed within the NBS framework, we set the alpha-level for the individual permutation test to *p* = 0.025.

In addition, we employed standard seed-based connectivity analyses as previously described^51^, using the DMN atlas regions as seeds and a mask comprising all DMN regions for second-level analysis. For details, see Supplementary Information.

### Behavioral testing and histology

Five days after scanning, rats underwent behavioral testing using an escapable foot shock paradigm^38^. Afterward, animals were killed for verification of virus expression and measurement of c-fos in the LHb as a marker of neuronal activity. See supplementary material for detailed methods and results.

## Results

### BOLD activity analysis

There was no significant group difference regarding BOLD activation during the laser block paradigm.

### Connectivity analyses

PPI did not reveal acute connectivity changes during the block laser sequence. Similarly, comparing the third and the first BOLD sequence (see Fig. 1), NBS yielded no significant group difference that would have indicated connectivity changes during continuous laser stimulation.

**Fig. 3:**
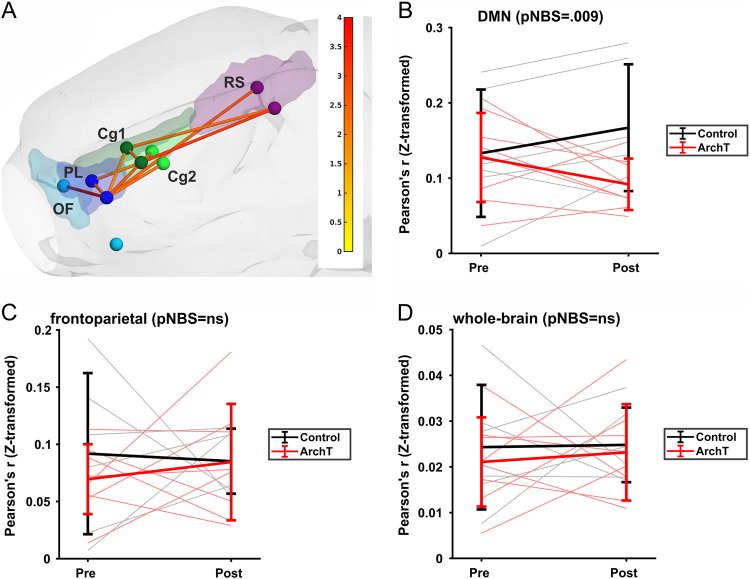
**a** Optogenetically reduced connectivity within the default-mode network in ArchT vs. control rats. Network-based statistics^50^ was used to test whether the longitudinal change (pre- vs. post-laser resting state) was different between ArchT and control group (*p* = 0.009). View is from left-anterior-superior. Colored spheres represent the DMN regions, and color-coding of the regions is equivalent to Fig. 2. Edges that are part of the significant network are indicated by orange/red lines, with the line color representing the T-value (see color bar on the right). **b** Quantitative representation of the connectivity changes in the DMN. Correlation coefficients were averaged over the network in order to display the group-by-time interaction effect. In the ArchT group, DMN connectivity significantly decreased following laser stimulation. Mean and standard deviation are shown, whereas individual correlation values are indicated in gray/pale red. For details, see main text. **c**, **d** Analogous analyses of an equally sized set of cortical regions **c** or the whole-brain network **d** did not reveal any group differences, suggesting the specificity of the DMN finding

Testing for delayed effects (by subtracting the first from the fourth sequence), we found a subnetwork showing a stronger connectivity decrease in the ArchT group (*p* = 0.009, all *p*-values refer to NBS permutation tests, one-sided, significance threshold *p* = 0.025). There was no opposite effect (i.e., no evidence for a stronger increase in ArchT group: *p* = 1.0). The significant network comprised eleven edges and nine nodes (right orbitofrontal cortex, bilateral prelimbic cortex, bilateral cingulate cortices 1 and 2 and bilateral retrosplenial cortex; see Figs. 3a, b). Post-hoc, a paired *T*-test within the ArchT group (post-laser vs. pre-laser) confirmed a significant decrease in connectivity (*p* = 0.014). In the control group, there was no significant change; if anything, the data showed a trend-level increase in connectivity (*p* = 0.04; significance threshold was *p* = 0.025 as NBS permutation test is one sided).

Next, to assess the specificity of our subnetwork and to exclude the possibility that the observed DMN connectivity decrease in the ArchT group was due to an unspecific global reduction in connectivity, we conducted the following additional analyses:

First, selecting a comparably sized network outside the DMN did not show analogous connectivity differences. Specifically, we performed an NBS analysis on a frontoparietal network, which—like the DMN network—consisted of five anatomically adjacent *cortical* regions per hemisphere (primary and secondary motor cortices, primary and secondary sensory cortices, parietal association cortex; *p* = .3, NBS permutation test; see Fig. 3c).

Second, an NBS analysis including all atlas regions (*n* = 92) did not reveal any significant or trend-level connectivity decrease (nor increase) on the whole-network level (*p* > 0.5, NBS permutation test). Correspondingly, we averaged correlation coefficients over the whole-brain correlation matrix (92 × 92), comparing them between groups (control vs. ArchT) and time (pre vs. post session). Confirming the specificity of our results, we could not detect neither a main effect of time or group, nor a group-by-time interaction effect (all *p*-values > 0.5)—see Fig. 3d.

Additional seed-connectivity analyses revealed altered connectivity between prefrontal and cingulate cortices (family-wise error corrected), both comparing pre vs. post-laser resting-state sessions, as well as pre vs. continuous laser stimulation. For details, see Supplementary Information.

### Cardiac frequency and head movement

In order to test whether anesthesia yielded stable physiological conditions, we compared heart rate between groups and sessions (and whether longitudinal change in heart rate might be different between the two groups)—this did not yield any differences. The same was true for head motion. See supplementary materials for details.

## Discussion

This is—to our knowledge—the first study to demonstrate a causal link between habenula modulation and DMN connectivity. We achieved this by using og-fMRI. Specifically, we detected a reduction in DMN connectivity following LHb perturbation with an inhibitory opsin in a rat model of treatment-resistant depression.

In human-depressive disorders, hyperconnectivity of the (especially anterior) DMN is one the most consistent neuroimaging findings^14–16^. Conversely, antidepressant administration leads to reduction in DMN connectivity, both in healthy volunteers^52^ and patients^53,54^. Thus, there is preliminary evidence that reduction of DMN connectivity is a neuroimaging correlate of antidepressant therapy.

On the other hand, downregulation of LHb activity appears as a promising therapeutic principle for treatment-resistant depression, based on both preclinical^6,55^ and preliminary clinical^12^ evidence. Our data suggest a connection between these two aspects of depression pathophysiology and treatment, although without demonstrating a specificity to depressive states, for lack of an analogous control experiment in wild-type animals.

According to a recent review^16^, it is the anterior DMN (medial prefrontal cortex, anterior cingulate cortex) that most consistently shows hyperconnectivity (in the sense of increased temporal correlation among the constituent regions) in resting-state studies of human depression^17–21^. Two recent meta-analyses also found DMN hyperconnectivity especially with the medial prefrontal cortex^14,15^, whereas hyperconnectivity with other regions (such as temporal or parietal regions) did not show consistent results between these two meta-analyses, probably due to differing inclusion criteria. Interestingly, hyperconnectivity within the anterior DMN was found to correlate with rumination^21^ and depression refractoriness^17^.

Hyperconnectivity within the posterior DMN has also been reported^17,19,20^, but less consistently^21^. Changes in connectivity between anterior and posterior DMN regions have been widely replicated (for review, see Mulders et al.^16^), although the direction of these changes probably differs between subregions: some studies showed increased connectivity in depression^28,29,31^, whereas others saw decreased connectivity^22–24^ or bidirectional changes^30^. When altered connectivity involving the posterior DMN was found, it concerned almost exclusively its medial portions, namely posterior cingulate cortex and adjacent precuneus^23,24,28–31^. The posterior cingulate cortex is considered homologous to the rat retrosplenial cortex^25,56^, which in turn is one of the central nodes in the rat DMN^25^.

Of note, we did not observe an acute effect on BOLD activity (as assessed during the 30-s block stimulation). This may be due to the inhibitory nature of the LHb modulation, which does not lead to immediate neuronal activation. Perhaps in correspondence to this, NBS did not reveal acute connectivity changes immediately *during* the laser stimulation (although such effects, spatially quite limited, were detected with the seed-based approach). Rather, the effects were delayed, such that connectivity alterations were detected during a second resting-state session *after* the laser stimulation. Speculatively, this suggests effects on brain plasticity, and is compatible with the functional neuroanatomy of the LHb: there is no direct connection between LHb and cortical areas. Rather, the LHb sends its main efferents to the monoaminergic nuclei (ventral tegmental area (VTA) and dorsal raphe nucleus), with mostly inhibitory effects^9–11^. The monoaminergic nuclei, in turn, have wide-ranging, modulatory efferents to subcortical and cortical areas. Rodent studies have begun to investigate the cortical downstream effects of the LHb-monoamine pathway: LHb neurons synapse primarily onto those VTA neurons that project to the mPFC^57^, and LHb inactivation leads to increased prefrontal dopamine release^58^. This pathway appears functionally relevant for avoidance behaviors^57^. In non-human primates, LHb and VTA show strongly opposite behavior in response to reward or punishment, with LHb inhibiting VTA^9^. The anterior cingulate cortex (part of the DMN) is also involved in monitoring negative reward prediction error and, speculatively, receives this information from the LHb^59^, perhaps via VTA. Finally, human neuroimaging data have suggested a modulation of DMN connectivity by both dopamine^60,61^ and serotonin^62,63^. This is in line with antidepressant drugs reducing DMN connectivity, as mentioned above^52–54^.

A better understanding of therapeutic mechanisms is urgently needed. In contrast to the case reports of LHb DBS cited above^12^, as well as some successful *open* DBS trials targeting other brain regions (reviewed in Morishita et al.^64^), the only two *double-blind* sham-controlled DBS studies yielded negative results^65,66^. This underscores the necessity to investigate the circuit dysfunctions in treatment-resistant depression, and how they are modified by treatment. Such an understanding would allow patient selection, monitoring of therapeutic response and refinement of treatment protocols^67^.

FMRI, unlike many other methods used in basic neuroscience, is a translational tool applicable in rodent models, non-human primates as well as in patients^68^. As mentioned above, recent high-resolution fMRI studies in humans have shown that the LHb is functionally connected with DMN regions^1,32^, and these connectivities were associated with depression scores^1^. This supports the potential clinical relevance of our findings. Naturally, these human data do not inform about causality, unlike the findings presented herein.

We also probed in an exploratory behavioral experiment whether a brief perturbation of a fraction of LHb neurons through ArchT inhibition would suffice to exert a relevant influence on behavior several days later, as predicted by pharmacologic LHb inhibition, where delayed, but not acute, effects on helplessness were observed^22^ (see Supplementary Methods). Although we observed a correlation between helpless behavior and LHb c-fos levels (as a marker of neuronal activity) within the ArchT group, we did not detect a group effect in our behavioral testing. One possible explanation might be that previous pharmacological inhibition of LHb^13^ had a longer duration of action. Future studies may need to probe different and in particular longer stimulation paradigms to identify an effective perturbation to influence behavior.

A further important consideration pertains to the fact that our study did not include analogous experiments assessing the effect of LHb perturbation in a non-depressed rat strain or other animal models, which means that we cannot show whether the mechanism demonstrated in this article operates outside the context of the animal model for depression we studied. Further studies of this kind are needed to test whether this effect is specific to certain animal models. Moreover, our data do not prove the depressive-like state of our sample, considering that stress exposure is needed in this animal model to cause a full-blown depressive phenotype^69^. We had chosen not to introduce further stress or perform behavioral testing *before* the MRI measurements, in order to minimize potential confounds, and considering that the animals had already been exposed to substantial stressors (e.g., repeated surgeries, single housing).

Additionally, apart from the escapable foot shock, other behavioral measures were not assessed, which might have revealed an effect of LHb perturbation. Also, we did not test behavior under concurrent laser stimulation. Future studies should include additional behavioral tests and assess acute vs. chronic effects of the optogenetic intervention on behavior.

Finally, a limitation of the NBS approach is the anatomical non-specificity of the findings, as the inference pertains to the cluster of significantly changing connections as a whole, rather than to individual region-of-interest (ROI) pairs. Our additional seed-based analysis yielded spatially much more restricted effects. However, the seed analysis also detected an immediate effect during the continuous laser stimulation, which was missed by NBS. This reflects the complementary strengths and weaknesses of seed-based vs. NBS analyses: whereas NBS is more sensitive to larger clusters of altered connectivity, seed-based approaches are more powerful for detecting localized changes.

Some further methodological aspects of our study deserve consideration: as we used a comparison group that underwent the same experimental protocol, our findings cannot be explained by laser artifacts, anesthesia duration, physiological changes or scanning-related stress. In particular, anesthesia is a highly relevant confounder, which needs to be adequately controlled for in functional connectivity studies. Medetomidine, which was used in our study, is associated with reduced cortical connectivity^70^. Importantly, we did not observe any group differences or longitudinal trends in potential confounds such as cardiac frequency (as a marker of arousal/anesthesia depth) or head motion, supporting the robustness of our findings.

It should be noted that only a part of all glutamatergic cells in the LHb were successfully transduced with the virus, thus limiting LHb perturbation to this sub-population. We did, however, not intend to completely abolish LHb function but rather reduce its activity, in order to model clinical interventions in humans. This is in accordance with recent data in the same animal model, where knock-down of calcium/calmodulin-dependent protein kinase type II (betaCaMKII, a synaptic scaffolding protein) reduced depressive-like behaviors, even at an infection rate of only around 20%^55^. The authors concluded that there was probably little redundancy in the LHb network, such that inhibition of a sub-population already has functional consequences.

Finally, it should be noted that in our study, we did not intend to assess functional connectivity of the LHb itself—such an analysis would have been unreliable due to fMRI artifacts around the tip of the optic fiber.

In summary, we employed og-fMRI as a translational tool, linking cellular neuroscience to neuroimaging phenotypes. Our study is the first to provide evidence for a causal relationship between LHb modulation and connectivity alterations in the DMN—two important factors of depression pathophysiology and, potentially, therapy.

## Electronic supplementary material

Supplementary Information(DOC 4505 kb)
