# Supplementary material for: Lateral habenula perturbation reduces default-mode network connectivity in a rat model of depression
Source: Transl Psychiatry. 2018 Mar 27;8:68. doi: 10.1038/s41398-018-0121-y (PMC5913319; doi:10.1038/s41398-018-0121-y)
Supplement: Supplementary file 1 — Supplementary Information(DOC 4505 kb) [file 41398_2018_121_MOESM1_ESM.doc]

# Supplementary Materials

**Lateral Habenula Perturbation Reduces Default-Mode Network Connectivity in a Rat Model of Depression**

C. Clemm von Hohenberg et al.

## Supplementary Methods

**Animals**

Three to four rats were housed per cage, kept in a standard 12-hour light/dark cycle and given food and water ad libitum.

**Virus**

AAV vectors were produced with AAV1/2 coat proteins and purified through heparin columns to a final viral concentration of approx. 10^16^ genome copies/ml. At 8 weeks of age, rats were anesthetized with isoflurane. 0.20 µl of purified virus was injected into the left and right lateral habenulae (from bregma: posterior 2.9 mm, lateral 1.7 mm with a 10° tilt such that the tip of the pipette pointed medially; ventral 4.8 mm from brain surface). Of the 19 rats used, 13 were injected with rAAV-CaMKIIa-ArchT:GFP, while the other 6 (control group) were injected with a virus lacking ArchT, and with a different fluorescent protein (rAAV-CaMKIIa-dTomato). Animals were assigned to receive either of the two viruses in an alternating way, without formal randomization. During the scanning procedures and preprocessing of imaging data, researchers were blind regarding the virus type.

**Implants**

Three weeks after virus injection, a fiber-optic cannula (core diameter 200 µm, numerical aperture 0.22, Thorlabs, Newton, New Jersey) was implanted stereotactically on either side. The same coordinates were used as for the injection, with the exception of the dorso-ventral coordinate, which was 4.4 mm from skull surface, to avoid damage to the LHb. Skull surface rather than brain surface was used for consistency because brain surface was more difficult to define reliably during the second surgery. The cannula was then fixed with dental cement.

**Laser stimulation**

For laser stimulation during fMRI acquisition, transistor-transistor logic (TTL) driven laser pulses were delivered from optical fibers (Thorlabs, Newton, New Jersey) coupled to a 25 mW, 593 nm, diode-pumped, solid-state laser (Shanghai Laser & Optics Century, Shanghai, China). For photostimulation during scanning, laser pulses were controlled via TTL modulation from a Master-8 (A.M.P.I., Jerusalem, Israel), which in turn was initially triggered by the first scanner pulse of each imaging sequence.

The laser power was set to 3 mW per hemisphere, as measured with the laser running continuously using a power meter (Thorlabs, Newton, New Jersey, USA). This power had been determined in preparatory experiments testing different laser powers: at 3 mW, heating artifacts were reliably absent (data not shown).

**Scanner**

A 94/20 Bruker Biospec MRI scanner (9.4 T; Bruker Ettlingen, Germany) with Avance III hardware, BGA12S gradient system with the maximum strength of 705 mT/m and running Paravision 5.1 software was used for all experiments. Transmission and reception were attained using a linear whole-body volume transmitter coil combined with an anatomically-shaped surface receive rat brain coil (with a hole for the optical fibers).

**Scanning procedures**

Rats were approximately eleven weeks of age at time of scanning. Animals were initially anaesthetized with 4 % isoflurane (Baxter Deutschland GmbH, Unterschleissheim, Germany) in a mixture of N2 (70 %) and O2 (30 %). The anesthesia was reduced to 2.5 % isoflurane for positioning in the scanner (head first, prone) and adjustments. Subsequently, a bolus of 0.08 mg/kg medetomidine (Domitor®, Janssen-Cilag, Neuss, Germany) was applied and the isoflurane tapered off in a stepwise manner. Ten minutes after the bolus, a continuous dose of 0.3 mg/kg/h medetomidine was started and continued throughout the fMRI experiments, which were started 30 minutes after bolus injection.

To reverse the sedative effect and compensate for the fluid loss during the experiments, Atipamezole (Antisedan®, Janssen-Cilag, Neuss; 5 mg/kg) and ~2 ml of saline were injected subcutaneously after the measurement.

During all measurements the animal body temperature was measured with a rectal probe and kept at 36 °C using warm water circulation pads. Breathing and cardiac rates were monitored using a respiration pad placed beneath the chest (Small Animal Instruments Inc., NY, USA) and a pulse oxymeter attached to the hind paw. Signals were recorded (10 ms resolution) using a signal breakout module (Small Animal Instruments Inc., NY, US).

In addition to standard localizer images, high-resolution T2-weighted 3D brain images were acquired (TR/TE_eff_ = 1200/50 ms, 192 x 225 matrix, field of view = 28.8 x 33.6 mm², 96 slices, 0.3 mm slice thickness) to achieve a robust normalization of the functional data to a digitized rat brain atlas.  B0-fieldmap images were recorded before every EPI sequence to correct for geometrical distortions (TR = 20 ms, TE (dual echo) = 1.7/5.7 ms, flip angle 29°, 64 x 64 x 64 matrix).

**Seed region analyses**

Seed-based analysis was performed as previously described (1). In brief, mean time courses of the DMN atlas regions were extracted from each animal using the unsmoothed EPIs; then data were smoothed by 0.6 mm full width at half maximum (FWHM) using SPM8. Correlation coefficients r were calculated between the atlas-extracted time courses and all voxels of the brain and afterwards transformed to z-scores using Fisher’s r-to-z transformation. These z-maps were subtracted between time points (e.g. post-laser resting state minus pre-laser resting state) and the resulting maps fed into a second level analysis (two sample t-test), masked to comprise all DMN regions. The threshold for significance was set to p<0.05, FWE corrected at the voxel level.

**Behavioral Testing**

The escapable foot shock paradigm has been described in detail elsewhere (2). It is the same paradigm that is applied for selecting rats during breeding of the CLH rat model used in this investigation. In brief, animals are exposed to electric foot shocks that can be stopped by pressing a lever. Fifteen subsequent trials are performed, each lasting 60 seconds maximum (if the animal fails to press the lever). For each session, the time is noted it takes the animal to press the lever (latency). If the animal fails to press the lever, this is counted as 60 s. For analysis, the following parameters can be used: sum of latencies of all fifteen trials, “failure pattern” (the number of trials where the animal fails to press the lever), and “delay pattern” (the number of trials where it takes the animal more than 20 s to press the lever).

We chose to perform the behavioral testing five days after the scanning, for the following reasons: On the one hand, we wanted to avoid additional stress confounding the imaging results; therefore the behavioral data were acquired *after* the scanning. On the other hand, the delay of five days was motivated by the following reasons: Firstly, we wanted to minimize the influence of surgery- and scanning-related stress and anesthesia on test performance. Secondly, we had previously reported that the effects of LHb inhibition on performance in this test were delayed by approximately one week (3). In order to increase the dose of LHb perturbation, we subjected animals to a second session of laser stimulation. This was done two days after scanning, i.e. three days before the behavioral testing. Animals were awake and freely moving (in a surrounding to which they had been well habituated) and received laser stimulation analogous to the stimulation during the third scanning session, repeated twice (i.e., 2*10 minutes stimulation at 30 Hz, pulse width 5 ms).

For statistical analyses, we used a non-parametric test (Mann-Whitney-U), since with the sample sizes used, normality cannot be assumed.

**Histology analysis**

100 minutes after the start of behavioral testing (range 92-123, matched between groups, p>.5), rats were perfused transcardially with PBS followed by perfusion with 4 % PFA, in deep isoflurane anesthesia. This time interval was chosen in order to measure c-fos as a marker of neuronal activity *during the behavioral testing* (see below). Brains were post-fixed in 4 % PFA for 24 h at 4 °C.

To determine the extent of fluorescent protein-tagged ArchT expression, sagittal slices (50 μm thickness) were cut with a vibratome. Sections were mounted with Fluoromount (Sigma) and analyzed using a Neurolucida System (Microbrightfield) attached to an epifluorescence microscope (Zeiss Imager, 20x objective).

To visualize c-fos expression in LHb, sections were incubated with a polyclonal rabbit antibody (1:10,000; Calbiochem) in blocking solution containing 0.25 % Triton X and 1 % bovine serum

albumin at 4°C overnight and subsequently with biotinylated secondary antibody and with peroxidase-avidin-biotin complex (both biotinylated goat anti-rabbit and ABComplex from Vector Laboratories) and visualized with 3,3’-diaminobenzidine (DAB). Per hemisphere, c-fos-immunoreactive cells were counted in serial sections of LHb and the area measured using the Neurolucida system (MBF Bioscience) with a 20× objective (Zeiss Imager). The mean density for each animal was calculated from sections of both hemispheres.

**Supplementary Results**

**Cardiac frequency**

Cardiac frequency was computed from the oxygen saturation measurements acquired during the MRI scanning. This was done using the Aztec (4) software employing the RETROICOR (5) method, which was also used for physiological noise correction during image preprocessing (see Methods section in main manuscript). As shown in Supplementary Figure S1, heart rate was reasonably stable over the whole measurement (around 4 Hz), although there was a slight increase in inter-individual variability. In the first two sessions, there was one animal with considerably higher pulse rate (around 6 Hz, see top left corner of graph), although this returned to normal values for the last two sessions. In order to exclude this as a confounder, we checked whether this animal (which belonged to the control group) was an outlier regarding Default-Mode Network connectivity. This was not the case (4^th^ rank among n=6 animals). Also, we repeated the main analysis of DMN resting-state connectivity changes, this time excluding this animal. All results were unchanged including the main finding (p=.016, NBS-corrected).

In order to further exclude any bias connected to cardiac frequency (which may also indicate different anesthesia/arousal levels), we compared heart rate (averaged over each session) between sessions and groups. There were no significant or trend-level differences between the two groups, nor between the sessions (non-parametric Kruskal-Wallis tests were used for all comparisons to account for the small sample size; all p-values>.1).

Lastly, the groups did not differ in the longitudinal heart rate changes between sessions (i.e. “group-by-session interaction”, p>.1). This is of importance since the main fMRI connectivity finding is also such an interaction effect.

**Supplementary Figure S1:** Cardiac frequency for each individual animal over all four scanning sessions. For clearer visualization, cardiac frequency was down-sampled (sampling frequency 0.2 Hz).
There is one outlier regarding heart rate during the first two sessions (heart rate around 6 Hz), although this returned to normal values during the last two sessions. For details, see text.

**Movement**

From the realignment step of the pre-processing pipeline, motion correction data were extracted for all six directions: three translation and three rotation directions. (It has to be noted though, that this parameter represents scanner drift more than physical head movement).

Subsequently, this data was differentiated, absolute values taken and summed up, to quantify total motion in each direction. The three translational directions and the three rotational directions were root-sum-squared, respectively, and the resulting values then compared between groups and sessions. (As for the cardiac data, non-parametric Kruskal-Wallis tests were used due to the small sample size.)

We wanted to test whether the main connectivity finding might be due to movement artifacts, which did not seem to be the case: First, there was no group difference in motion values. Second, there was no difference between the pre-laser and post-laser resting state sessions. Third, there was no group-by-session interaction effect (i.e. there was no group difference regarding the longitudinal change of motion between these sessions). All of this was tested both for translational and rotational motion (all p>.1).

**Seed correlation analyses**

Comparing groups regarding the continuous-laser minus pre-laser difference, seed-connectivity analysis detected an effect between cingulate cortex 2 (Cg2) right (seed) and a small region within the orbitofrontal cortex left. Regarding the post-laser minus pre-laser contrast, there was an effect between right prelimbic (PL) cortex (seed) with a small cluster within the ipsilateral and contralateral PL and contralateral Cg1 and Cg2.

| **Time contrast** | **Seed region** | **Direction of effect** | **T** | **P (FWE, peak level)** | **Cluster size, p<.05(FWE)/ p<.001(uncorr.)** | **Peak voxel (Paxinos space)** | | | **regions** | **Legend Fig. S2** |
| --- | --- | --- | --- | --- | --- | --- | --- | --- | --- | --- |
|  |  |  |  |  |  | **X** | **Y** | **Z** |  |  |
| “pre vs. continuous” | Cg2 r | Stronger decrease in ArchT | 6.65 | .021 | 1 / 2 | -6 | 47 | -36 | OF l | A |
| “pre vs. post” | PL r | Stronger decrease in ArchT | 6.36 | .032 | 1 / 3 | 2 | 25 | -36 | PL l, PL r, | B |
|  |  |  | 6.01 | .054 (trend) | 1 / 4 | -17 | 18 | -29 | Cg1 l, Cg2 l | C |

**Supplementary Table S1:** Seed-based connectivity analysis. Abbreviations: l, left; r, right; Cg2, cingulate cortex 2; PL, prelimbic cortex; OF, orbitofrontal cortex; “pre”, pre-laser resting state; “continuous”, BOLD acquisition under continuous laser stimulation; “post”, post-laser resting state.


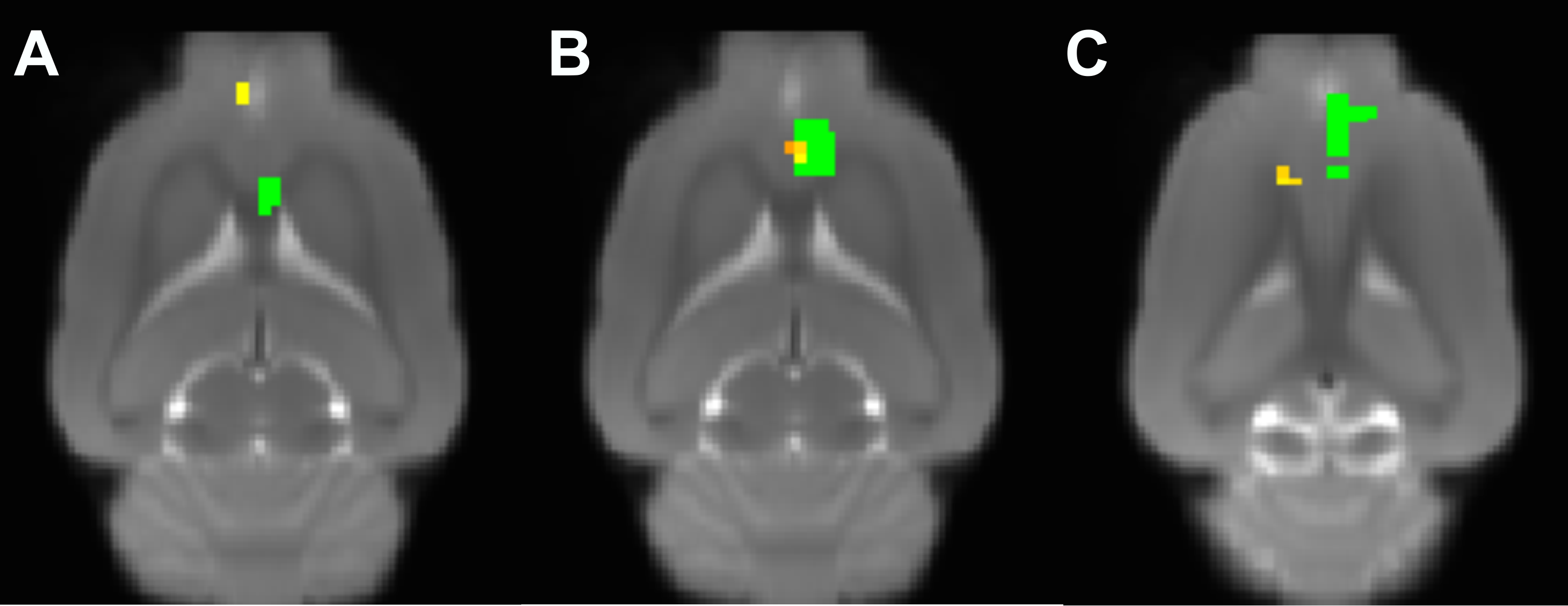


**Supplementary Figure S2:** Seed-based connectivity analysis revealing differential connectivity changes in DMN (stronger connectivity decrease in the ArchT group), both comparing “pre-laser” vs. “continuous stimulation” (A) and “pre-laser” vs. “post-laser” (B and C). See Table S2, rightmost column, for legend of the significant clusters shown. All clusters are p<.05, FWE-corrected. For clarity, also surrounding voxels with p<.001, uncorrected, are shown. The seed region is shown in green.

**Descriptive connectivity maps of the two groups individually**

**Supplementary Figures S3-S22:** Descriptive connectivity maps of the two groups during continuous laser stimulation as well as during the “post-laser” resting state. Green: seed regions (identical DMN regions as illustrated in the main manuscript: prelimbic cortex, orbitofrontal cortex, cingulated cortices 1 and 2, retrosplenial cortex). Blue: seed correlation in the control group; red: seed correlation in the ArchT group; violet: overlap. There is a tendency toward less extensive connectivity in the ArchT group, with some regions also showing the opposite effect. Threshold for display is p<0.05, cluster size k>20.


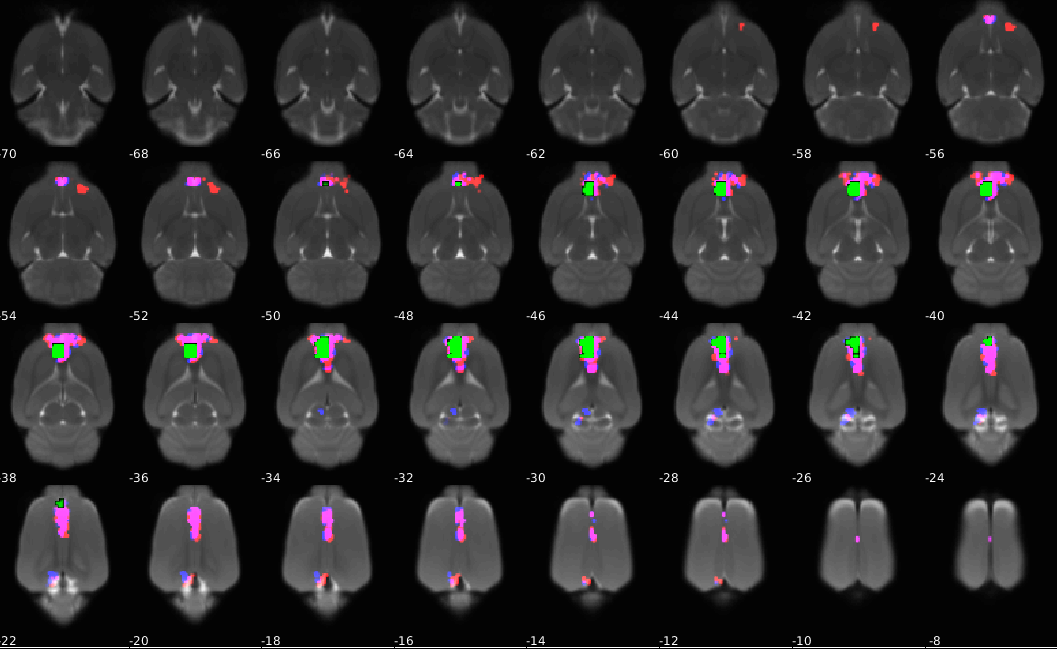


**Figure S3:** connectivity during continuous laser stimulation. Seed: prelimibic cortex left.


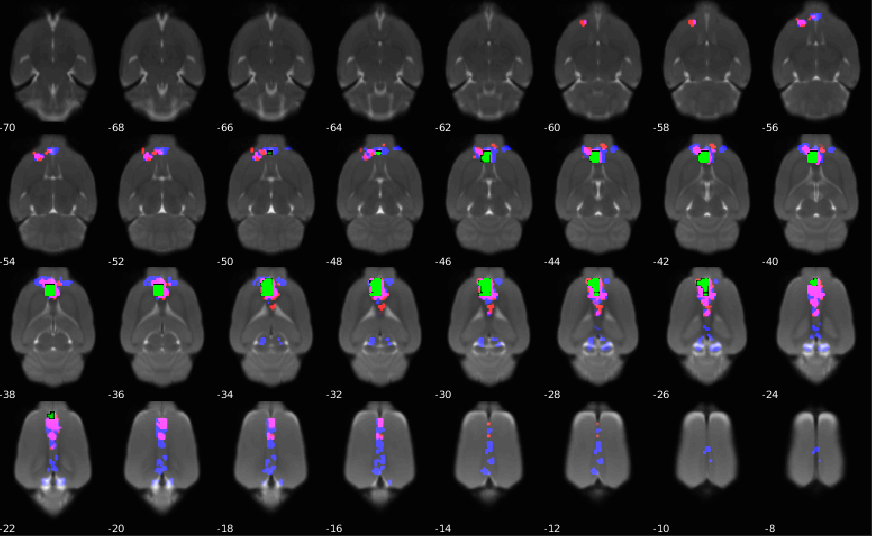


**Figure S4:** connectivity post-laser resting state. Seed: prelimibic cortex left.


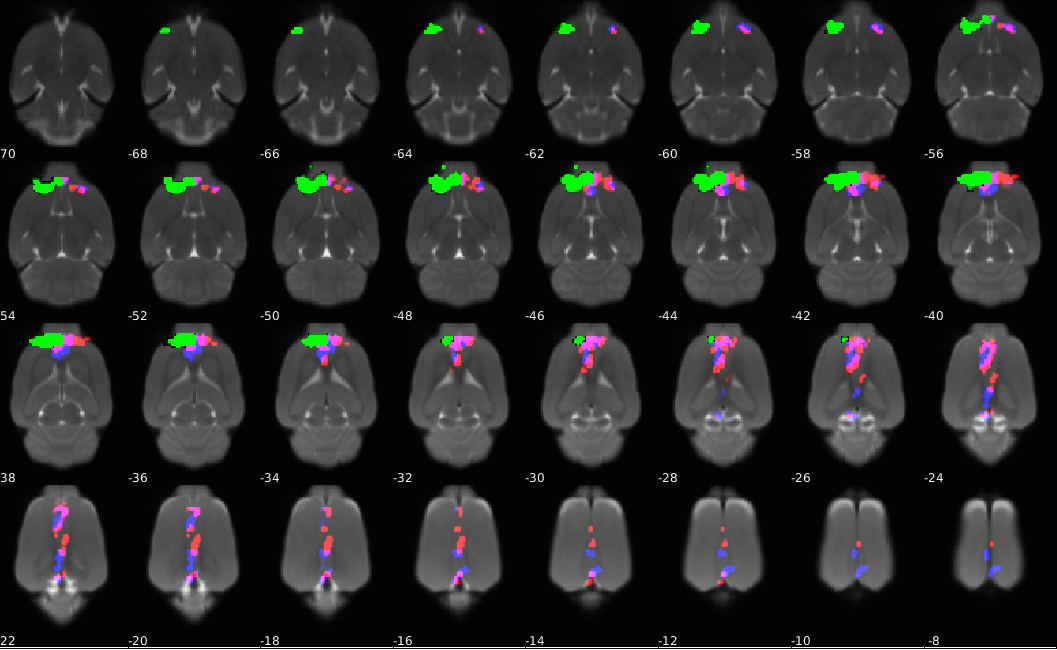


**Figure S5:** connectivity during continuous laser stimulation. Seed: orbitofrontal cortex left.


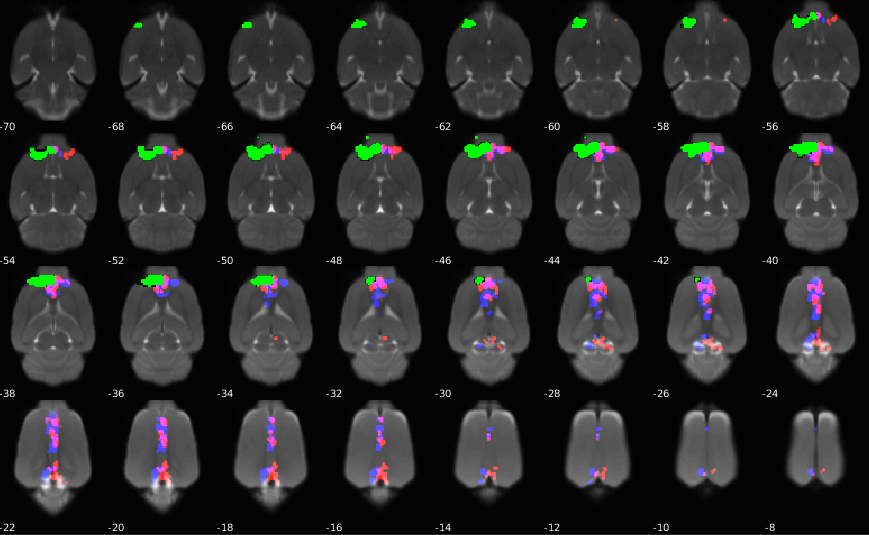


**Figure S6:** connectivity post-laser resting state. Seed: orbitofrontal cortex left.

**
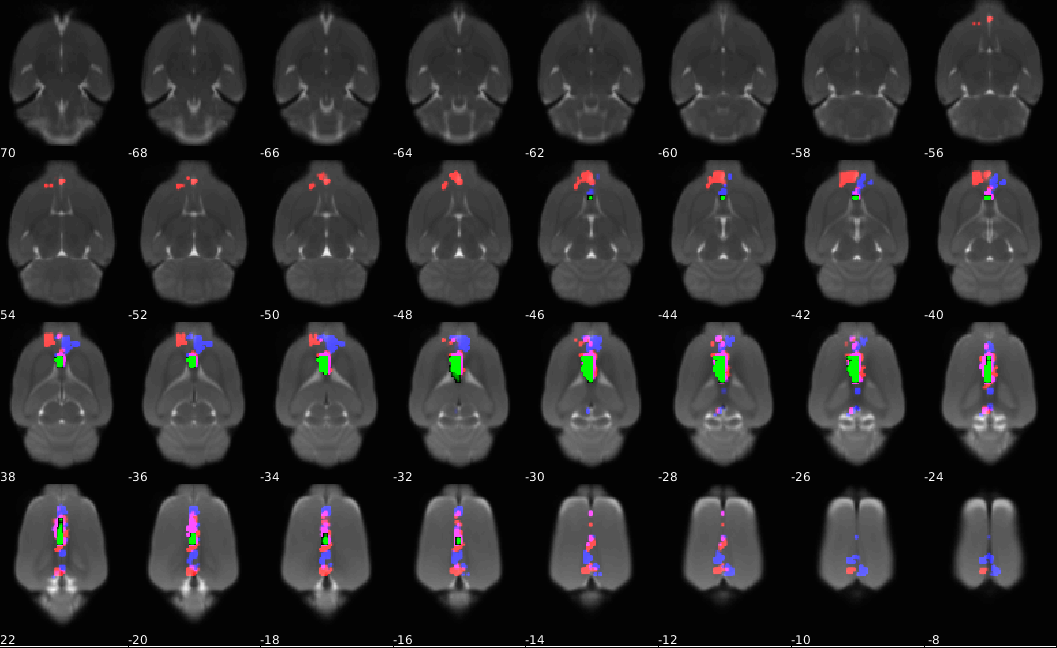
**

**Figure S7:** connectivity during continuous laser stimulation. Seed: cingulate cortex 2 left.


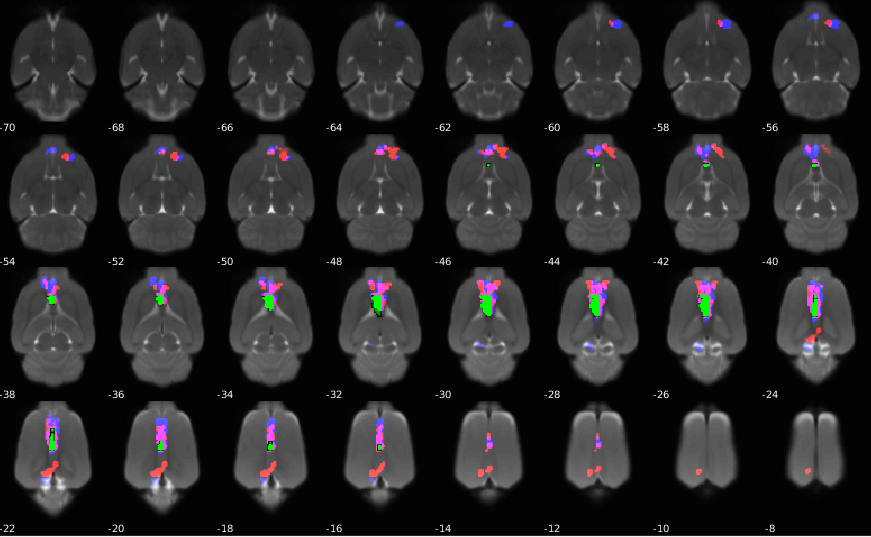


**Figure S8:** connectivity post-laser resting state. Seed: cingulate cortex 2 left.


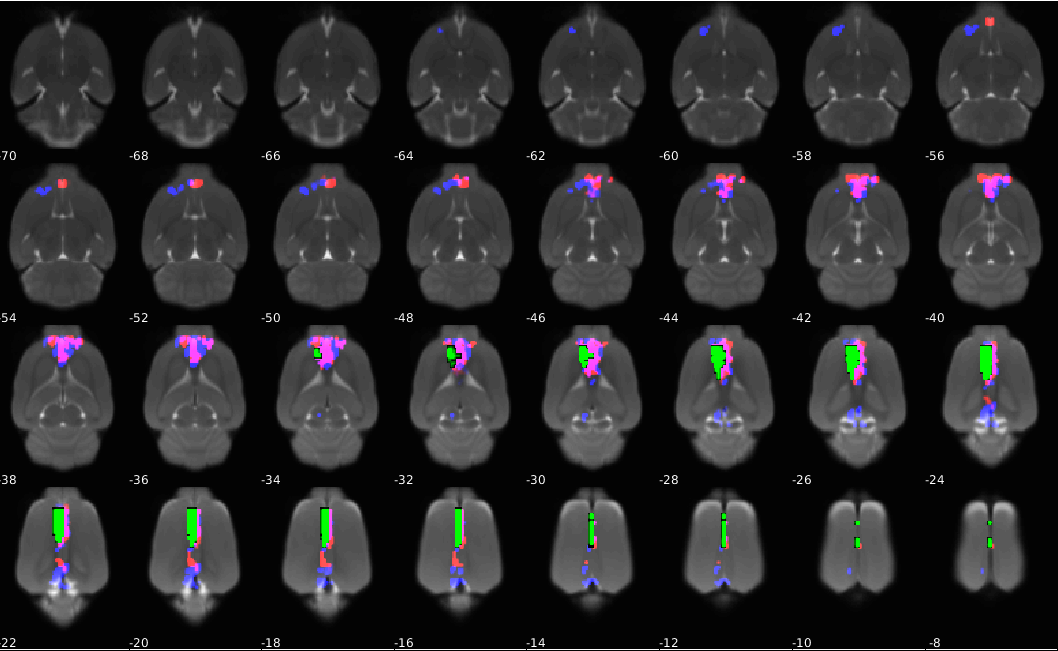


**Figure S9:** connectivity during continuous laser stimulation. Seed: cingulate cortex 1 left.


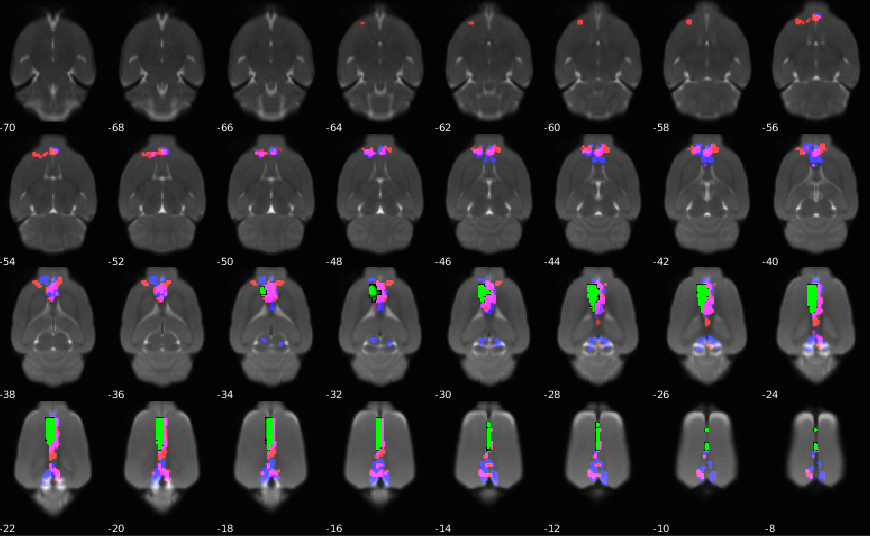


**Figure S10:** connectivity post-laser resting state. Seed: cingulate cortex 1 left.

**
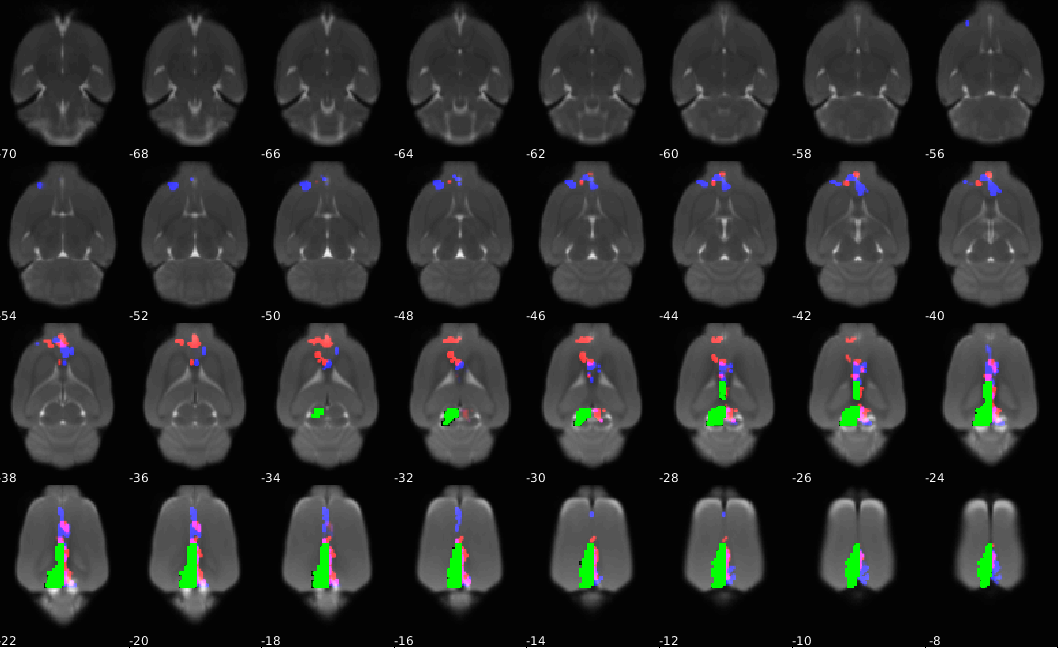
**

**Figure S11:** connectivity during continuous laser stimulation. Seed: restrosplenial cortex left.


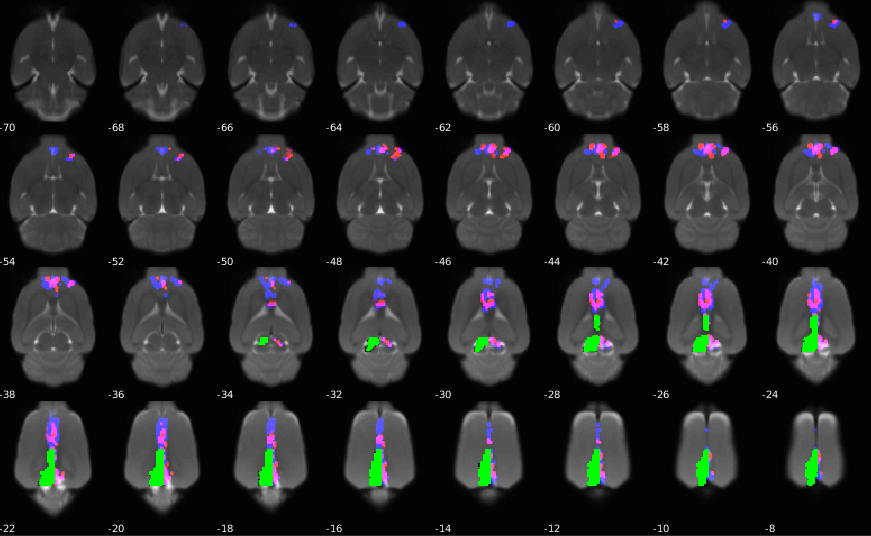


**Figure S12:** connectivity post-laser resting state. Seed: restrosplenial cortex left.


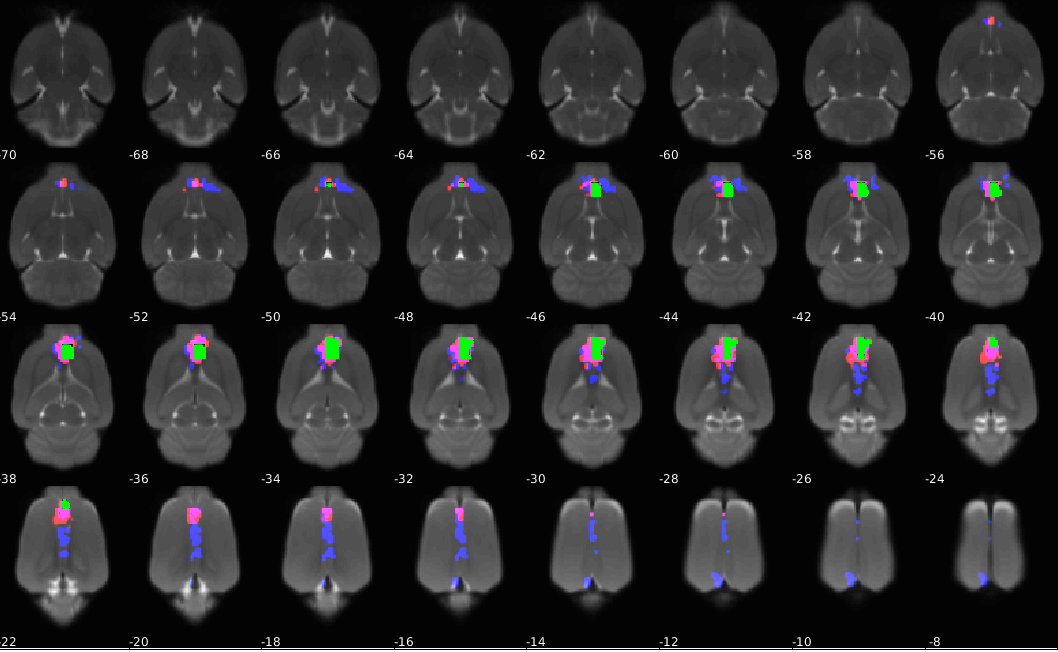


**Figure S13:** connectivity during continuous laser stimulation. Seed: prelimibic cortex right.

**
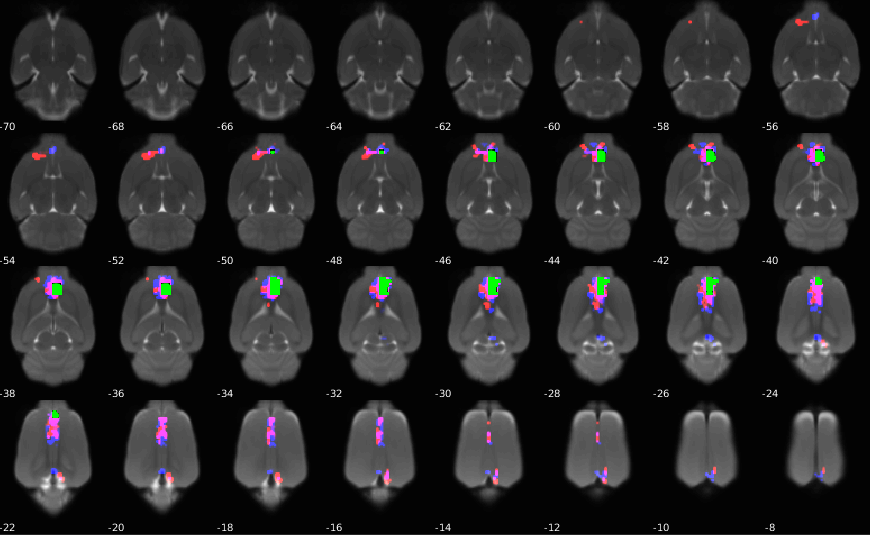
**

**Figure S14:** connectivity post-laser resting state. Seed: prelimibic cortex right.


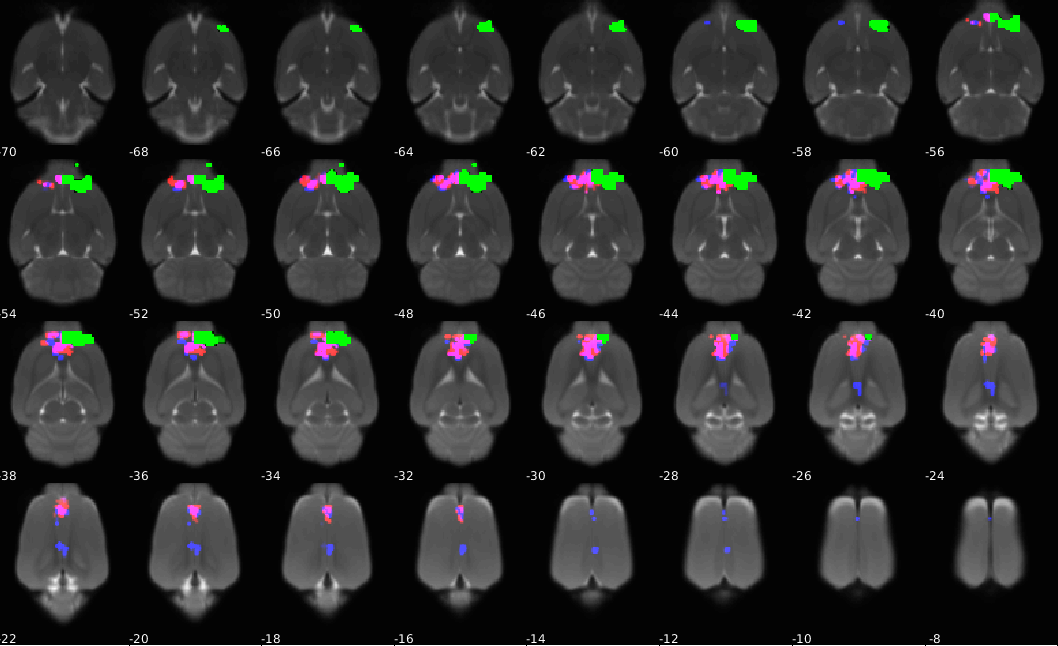


**Figure S15:** connectivity during continuous laser stimulation. Seed: orbitofrontal cortex right.

**
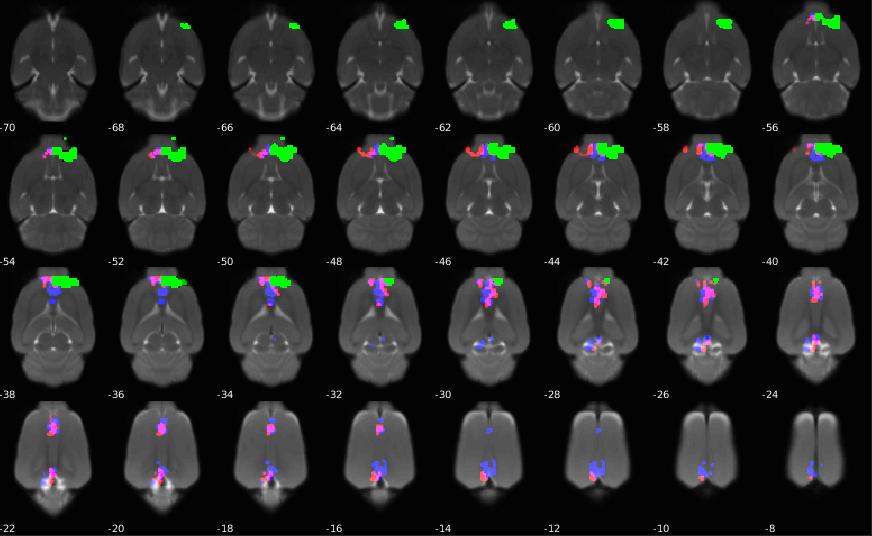
**

**Figure S16:** connectivity post-laser resting state. Seed: orbitofrontal cortex right.

**
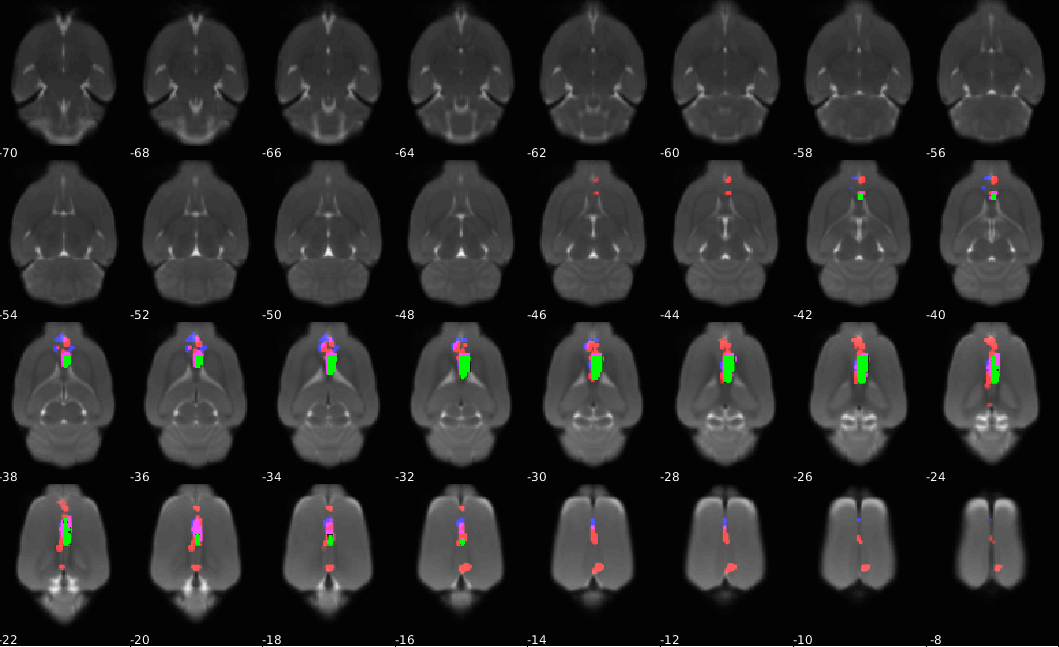
**

**Figure S17:** connectivity during continuous laser stimulation. Seed: cingulate cortex 2 right.

**
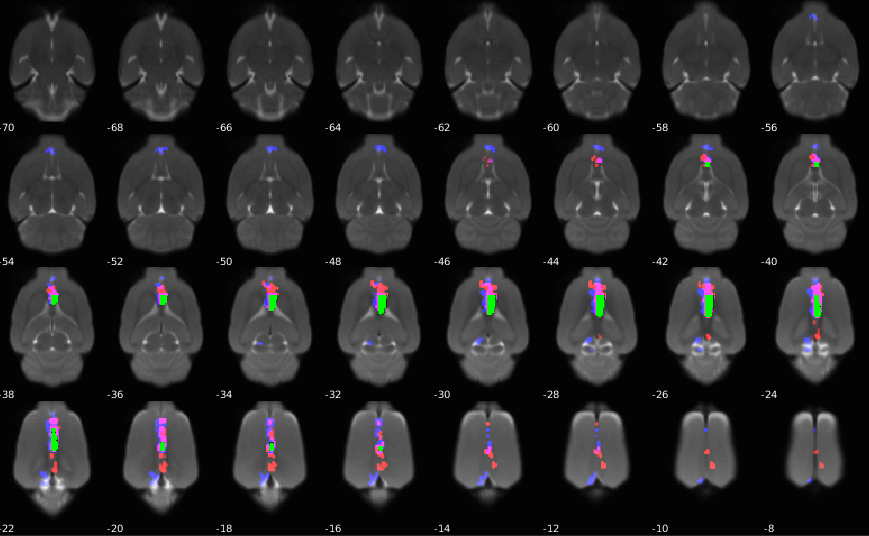
**

**Figure S18:** connectivity post-laser resting state. Seed: cingulate cortex 2 right.

**
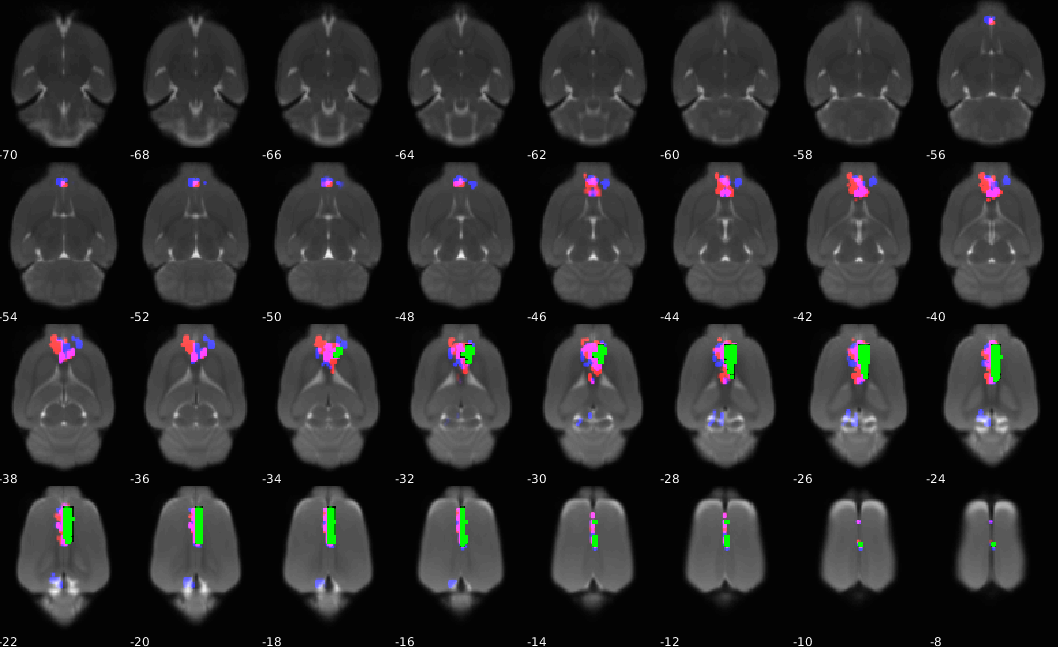
**

**Figure S19:** connectivity during continuous laser stimulation. Seed: cingulate cortex 1 right.

**
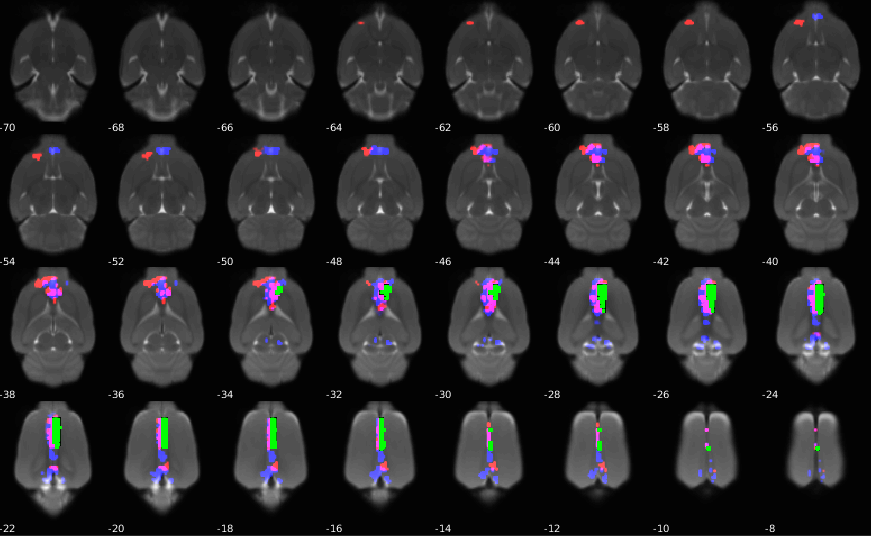
**

**Figure S20:** connectivity post-laser resting state. Seed: cingulate cortex 1 right.


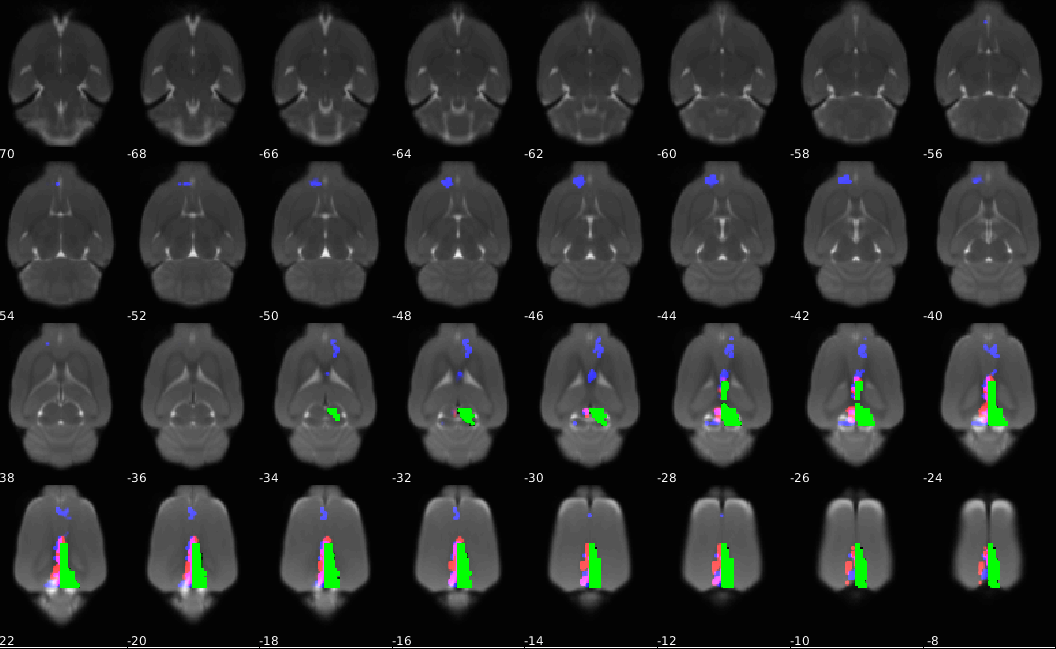


**Figure S21:** connectivity during continuous laser stimulation. Seed: retrosplenial cortex right.

**
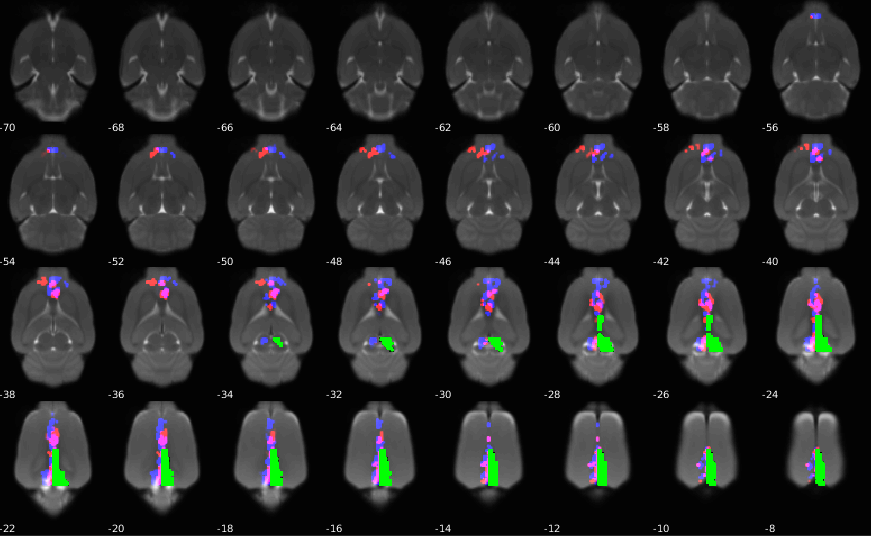
**

**Figure S22:** connectivity post-laser resting state. Seed: retrosplenial cortex right.

**Behavioral Testing**

There were no significant group differences.

| **Parameter** | **Control** | **ArchT** | **P** |
| --- | --- | --- | --- |
| **Sum of Latencies** [sec] | 580.5 | 548.5 | MWU=23; p=.9 |
| **Failure Pattern** [number of trials] | 7 | 6.6 | MWU=23.5; p=.9 |
| **Delay Pattern** [number of trials] | 10 | 9.9 | MWU=23.5; p=.9 |

**Supplementary Table S2.** Delayed behavorial testing with the escapable foot shock paradigm did not yield a significant group difference. Abbreviations: MWU: Mann-Whitney-U.

**c-Fos expression**

c-Fos data were available for all 8 ArchT animals and 5 (out of 6) control animals. There was no significant group difference in c-Fos levels in LHb (Mann-Whitney-U=15, p=.5).

In an exploratory analysis, we assessed correlations between LHb c-Fos and behavioral performance (sum of latencies). Within the ArchT group, we found such an association (Spearman’s rho=.74, p=.04, two-sided, see Supplementary Figure 1). In the control group, there was no correlation (Spearman’s rho=-.30, p=.6). The correlation within the ArchT group may reflect different levels in the efficacy of the inhibitory optogenetic manipulation that changed circuit function still after several days, reflected by lowered c-fos levels and reduced helplessness in the behavioral testing.

When interpreting these findings and comparing them to the neuroimaging results, it needs to be considered that the time scales of potential treatment effects differed substantially between the fMRI measurements (minutes) and the behavioral testing (days).

**Supplementary Figure S23.** In an exploratory analysis, c-Fos in LHb correlated with helpless behavior in the ArchT group (Spearman’s rho=.74, p=.04, two-sided), but not in the control group.

**Supplementary Literature**

1. Gass N. Sub-Anesthetic Ketamine Modulates Intrinsic BOLD Connectivity within the Hippocampal-Prefrontal Circuit in the Rat. Neuropsychopharmacology. 2013.

2. Vollmayr B, Henn FA. Learned helplessness in the rat: improvements in validity and reliability. Brain Res Brain Res Protoc. 2001; 8(1): 1-7.

3. Winter C, Vollmayr B, Djodari-Irani A, Klein J, Sartorius A. Pharmacological inhibition of the lateral habenula improves depressive-like behavior in an animal model of treatment resistant depression. Behavioural brain research. 2011; 216(1): 463-5.

4. van Buuren M, Gladwin TE, Zandbelt BB, van den Heuvel M, Ramsey NF, Kahn RS, et al. Cardiorespiratory effects on default‐mode network activity as measured with fMRI. Human brain mapping. 2009; 30(9): 3031-42.

5. Glover GH, Li TQ, Ress D. Image‐based method for retrospective correction of physiological motion effects in fMRI: RETROICOR. Magnetic Resonance in Medicine. 2000; 44(1): 162-7.
